# The Transcription Factor *AtDOF4.7* Is Involved in Ethylene- and *IDA*-Mediated Organ Abscission in *Arabidopsis*

**DOI:** 10.3389/fpls.2016.00863

**Published:** 2016-06-17

**Authors:** Gao-Qi Wang, Peng-Cheng Wei, Feng Tan, Man Yu, Xiao-Yan Zhang, Qi-Jun Chen, Xue-Chen Wang

**Affiliations:** ^1^State Key Laboratory of Plant Physiology and Biochemistry, College of Biological Sciences, China Agricultural UniversityBeijing, China; ^2^Rice Research Institution, AnHui Academy of Agricultural SciencesHefei, China; ^3^Department of Food and Biological Technology, College of Food Science and Nutritional Engineering, China Agricultural UniversityBeijing, China

**Keywords:** abscission, abscission zone, DOF, ethylene, IDA, MAPK, phosphorylation

## Abstract

Organ abscission is an important plant developmental process that occurs in response to environmental stress or pathogens. In *Arabidopsis*, ligand signals, such as ethylene or *INFLORESCENCE DEFICIENT IN ABSCISSION (IDA)*, can regulate organ abscission. Previously, we reported that overexpression of *AtDOF4.7*, a transcription factor gene, directly suppresses the expression of the abscission-related gene *ARABIDOPSIS DEHISCENCE ZONE POLYGALACTURONASE 2 (ADPG2)*, resulting in a deficiency of floral organ abscission. However, the relationship between *AtDOF4.7* and abscission pathways still needs to be investigated. In this study, we showed that ethylene regulates the expression of *AtDOF4.7*, and the peptide ligand, *IDA* negatively regulates *AtDOF4.7* at the transcriptional level. Genetic evidence indicates that *AtDOF4.7* and *IDA* are involved in a common pathway, and a MAPK cascade can phosphorylate AtDOF4.7 *in vitro*. Further *in vivo* data suggest that AtDOF4.7 protein levels may be regulated by this phosphorylation. Collectively, our results indicate that ethylene regulates *AtDOF4.7* that is involved in the *IDA*-mediated floral organ abscission pathway.

## Introduction

Plant floral organ abscission is an important developmentally controlled process. *Arabidopsis thaliana* is an ideal model in which to study organ abscission (Bleecker and Patterson, 1997). In *Arabidopsis*, at the bases of the filaments, petals, and sepals in the floral organ, there are some small, high-density cell layers known as abscission zone (AZ), where the abscission process takes place (Patterson, 2001). The AZ cells perceive the abscission signal and subsequently activate cell wall-degrading proteins. Finally, the pectin-rich middle lamellae of AZ cell walls are dissolved, resulting in the detachment of unwanted organs from the main plant body (Patterson, 2001; Patterson and Bleecker, 2004; Niederhuth et al., 2013a).

Ethylene is thought to temporally regulate floral organ abscission (Jackson and Osborne, 1970; Niederhuth et al., 2013a). Historically, exogenous ethylene treatment has been shown to cause acceleration of abscission (Patterson and Bleecker, 2004; Butenko et al., 2006). Several key components of ethylene signaling, including *ETHYLENE RESPONSE 1 (ETR1) and ETHYLENE INSENSITIVE 2 (EIN2)*, have been found to participate in the regulation of floral organ abscission in *Arabidopsis* (Chang et al., 1993; Patterson and Bleecker, 2004; Butenko et al., 2006). Both the *etr1* and *ein2* ethylene-insensitive mutants exhibit significantly delayed abscission.

Several reports have indicated that abscission can be regulated in an ethylene-independent manner. It was originally found that the important plant hormone auxin could regulate the differentiation of the AZ of the leaf rachis in *Sambucus nigra* through transportation of auxin from the organ distal to the AZ (Osborne and Sargent, 1976; Morris, 1993). In *Arabidopsis*, auxin negatively regulates several polygalacturonases (*PGs*) in the dehiscence zone (DZ) to cause a delay in cell separation (Dal Degan et al., 2001; Ellis et al., 2005). Mutation of *Auxin Response Factor 2 (ARF2)* can lead to a delay in floral organ abscission. Furthermore, the effect of a delay in abscission can be enhanced by mutations in *ARF1, ARF2, NPH4/ARF7*, and *ARF19* (Ellis et al., 2005; Okushima et al., 2005), suggesting that these overlapping genes regulate auxin-mediated floral organ abscission in an ethylene-independent manner.

Other than phytohormone, signaling peptides also play important roles in the regulation of abscission. In *Arabidopsis*, a short peptide, INFLORESCENCE DEFICIENT IN ABSCISSION (IDA) has a key role in the regulation of floral organ abscission (Butenko et al., 2003; Liu et al., 2013). The *ida* single mutant fails to abscise its floral organs (Butenko et al., 2003), and constitutive expression of *IDA* in *Arabidopsis (35S:IDA)* induces premature organ abscission (Stenvik et al., 2006). Two leucine-rich repeat receptor-like protein kinases (LRR-RLKs), HAE and HSL2, are required for organ abscission. Double mutants for these two genes display organ abscission defects (Jinn et al., 2000; Cho et al., 2008; Stenvik et al., 2008; Niederhuth et al., 2013b). As previously reported, *Arabidopsis* perceives and responds to environmental signals through an interaction between extracellular peptides and plasma membrane-bound RLKs (Stenvik et al., 2008). IDA may act as a ligand binding to the HAE/HSL2 RLK, which transmits the abscission signal to downstream substrates to initiate organ abscission (Cho et al., 2008; Stenvik et al., 2008).

Mitogen-activated protein kinase (MAPK) cascades play crucial roles in regulating various plant responses. There are two MEKs (MKK4 and MKK5) that are involved in floral organ abscission in *Arabidopsis* (Cho et al., 2008). Their downstream target substrates are MPK3 and MPK6 (Ren et al., 2002; Cho et al., 2008). *MKK4* and *MKK5* RNA interference (RNAi) transgenic lines show pleiotropic effects, including organ abscission deficiency, and constitutively active mutants of MKK4^DD^ (T224D/S230D) and MKK5^DD^ (T215D/S221D) that strongly activate endogenous MPK3 and MPK6 can restore abscission in the *ida-2* and *hae hsl2* mutants (Ren et al., 2002; Cho et al., 2008). Although the *mpk3* and *mpk6* single mutants exhibit normal organ abscission, the *mpk3 mpk6* double mutant is lethal (Wang et al., 2007), while functionally inactive forms of *MPK6* are expressed in *mpk3* mutant plants, *mpk3/MPK6*^KR^ (Lys, the key residue of phosphate transferring, mutated to Arg) or *mpk3/MPK6*^AF^ (the conserved sites of phosphorylation by MKK, Thr and Tyr, mutated to Ala and Phe, respectively), survive and display loss of organ abscission (Cho et al., 2008). Furthermore, MPK6 has reduced kinase activity in *hae hsl2* and *ida-2* mutants. These results demonstrate that MPK6, but not MPK3, plays a dominant role in the regulation of floral organ abscission (Cho et al., 2008). In the ethylene-independent abscission pathway, IDA couples with HAE and HSL2, activating the downstream MAPK cascade to phosphorylate the substrates and initiate the separation of the AZ cells (Cho et al., 2008; Shi et al., 2011; Niederhuth et al., 2013a,b).

Generally, ethylene regulates the timing of floral organ abscission, while *IDA* influences the degree of abscission. The physiological process of floral organ abscission is generally divided into four stages: (1) differentiation and formation of the AZ; (2) transduction of abscission signals; (3) activation of the abscission process; and (4) post-abscission transdifferentiation (Patterson, 2001; Niederhuth et al., 2013a). The second and third stages of abscission, in which ethylene, IDA, HAE/HSL2, MAPKs and PGs play important roles, have been well-described (Niederhuth et al., 2013a). However, further investigation is needed to understand how these two stages are linked.

We previously reported that AtDOF4.7, a member of the *Arabidopsis* DNA binding with one finger (DOF) transcription factor family, functions as an abscission inhibitor to directly regulate the expression of *ADPG2*, which encodes a cell wall-hydrolyzing enzyme, to initiate cell separation (Wei et al., 2010). Nevertheless, it is still an open question whether *AtDOF4.7* is a factor that acts between the second and third stage of abscission, potentially in a cascade of abscission signals that are transmitted from the second to the third stage through *AtDOF4.7*. In the present study, we demonstrated that *AtDOF4.7* is an additional component of the *IDA*-mediated abscission pathway, and that it is regulated by ethylene and *IDA*. Our results suggest that *AtDOF4.7* is regulated by both the ethylene-dependent and ethylene-independent pathways.

## Materials and Methods

### Plant Material

*Arabidopsis thaliana* (Columbia-0 ecotype) plants were grown at 22°C in a growth chamber under a 16 h light/8 h dark photoperiod and a light intensity of 120 μmol m^-2^ s^-1^.

The *Promoter_AtDOF4.7_::GUS* line (Wei et al., 2010) was crossed with *ein2-1, etr1-1*, and *ida-2* (SALK_133209), and *35S:AtDOF4.7 (S107;* Wei et al., 2010) was crossed with *35S:IDA* and *GVG-MKK5^DD^* (Ren et al., 2002). The sequencing primers used to verify hybrids are presented in online Supplementary Table S1. Kanamycin (50 μg ml***^-^***^1^) was employed to select the *35S:IDA* and *ida-2* mutant plants (Butenko et al., 2003; Stenvik et al., 2006). Hygromycin B (25 μg ml***^-^***^1^) was used to select plants overexpressing *AtDOF4.7*.

The locus codes of all genes investigated or discussed in this article are listed below: *AtDOF4.7, At4g38000; EIN2, At5g03280; ETR1, At1g66340; IDA, At1g68765; HAE, At4g28490; HSL2, AT5G65710; MKK4, At1g51660; MKK5, At3g21220; MPK3, At3g45640; MPK6, At2g43790; ACTIN2, At3g18780;* and *ADPG2, AT2G41850.*

### Ethylene and DEX Treatments

*Promoter_AtDOF4.7_::GUS/ein2-1* seeds were germinated on 1/2 Murashige and Skoog (MS) medium containing 5 μM ACC in the dark for 3 days to select for ethylene-insensitive mutants.

To analyze the expression pattern of *AtDOF4.7* in response to ethylene, 4-weeks-old *Promoter_AtDOF4.7_::GUS* plants were maintained in an air-tight growth chamber with or without ethylene at 10 ppm (10 μl l^-1^) for 3 days prior to the analysis.

To observe the abscission phenotype of *S107/MKK5^DD^*, siliques of *S107/MKK5^DD^* plants were treated with or without 0.02 μM DEX (Sigma, USA) for 24 h to induce *MKK5^DD^* expression. The siliques were then photographed with a Cannon G12 camera. Detached leaves from *S107/MKK5^DD^* plants were immersed in 15 μM DEX for various times prior to western blotting. Each treatment was repeated at least three times.

### β-Glucuronidase (GUS) Assay

β-Glucuronidase (GUS) gene expression was analyzed by staining different flower positions along the inflorescence as described previously (Wei et al., 2010). Histochemical GUS staining in the AZ cells of siliques was observed with an Olympus SZX16-DP72 stereo microscope system.

### Quantitative Real-Time and Semi-quantitative RT-PCR

For semi-quantitative RT-PCR analysis, total RNA samples were extracted from *Arabidopsis* siliques using the RNeasy^®^ Plant Mini Kit (Qiagen, Germany), and the RNA was reversely transcribed into cDNA with a Reverse Transcription System (Promega, USA). Semi-quantitative RT-PCR assays were run in a MJ Mini Personal Thermal Cycler (Bio-Rad, USA) with 25 thermal cycles. *ACTIN2* (*At3g18780*) was employed as an internal control.

For quantitative real-time RT-PCR (qRT-PCR) analysis, total RNA was extracted from whole flowers and siliques, and qRT-PCR assays were run using the 7500 Real-Time PCR System (ABI, USA). After normalization to an internal control (*ACTIN2*; Charrier et al., 2002), the relative levels of gene expression were calculated via the Delta-Delta Ct (2^-ΔΔCt^) method (Livak and Schmittgen, 2001). All primers are given in online Supplementary Table S2. Each relative gene expression assay was repeated at least three times.

### Yeast Two-Hybrid Experiment and BiFc

A yeast two-hybrid (Y2H) experiment was performed as described previously (Wei et al., 2010). Cells that had been co-transformed with the *pAD-GAL4-AtDOF4.7* (Wei et al., 2010) and *pBD-GAL4-MPK3/MPK6* constructs (Xu et al., 2008) were cultured and transferred to 5 mM 3-AT medium lacking Trp, Leu, His, and Ade, followed by incubation at 28°C for 3 days. Living yeast colonies were photographed with a Cannon G12 camera.

The bimolecular fluorescence complementation (BiFc) assay (Walter et al., 2004), co-transformation of *pUC-SPYCE-MPK3/MPK6* and *pUC-SPYNE-AtDOF4.7* (Miao et al., 2006) and examination of YFP fluorescence were all performed as described previously (Walter et al., 2004).

### Preparation of Recombinant Proteins

*Escherichia coli* cells (strains BL21 and DE3) were transformed with the *MPK3/6*-pET30a(+)-His and *MKK5^DD^*-pET28a(+)-FLAG constructs and then incubated as described previously (Liu and Zhang, 2004). His-tagged MPK3/6 recombinant proteins and FLAG-tagged MKK5^DD^ recombinant proteins were purified as described elsewhere (Xu et al., 2008). The *AtDOF4.7* CDS was cloned into pMal-c2x (NEB, USA) in frame with the N-terminal MBP tag, and the construct was transformed into the *E. coli* strain Rosetta (DE3). Recombinant protein expression was induced with 0.1 mM isopropylthio-β-galactoside (IPTG) for 8 h at 18°C. The MBP-tagged protein was purified using amylose resin (NEB, USA) according to the manufacturer’s instructions.

### Protein Extraction and Western Blotting Assay

Total protein was extracted from *Arabidopsis* silique tissues as described previously (Liu et al., 2007). The protein concentration was determined with a Bio-Rad protein assay kit (Bio-Rad, USA), using bovine serum albumin (BSA) as the standard. For western blotting assay, total protein extracts from each sample (10 μg per gel lane) were separated on 12% SDS-polyacrylamide gels. After electrophoresis, the proteins were electro-transferred to PVDF membranes (Millipore, USA). The membranes were subsequently incubated with an anti-Flag antibody (Cell Signaling, 1:10,000 dilution), washed, and then incubated with HRP-conjugated goat anti-rabbit IgG as a secondary antibody (1:10,000 dilution). The resultant protein bands were visualized by treating the membrane with the Immobilon^TM^ Western Chemiluminescent HRP Substrate (Millipore, USA), following the manufacturer’s instructions. Bands intensities in the western blots were compared using ImageJ software.

### Phosphorylation of AtDOF4.7 *In Vitro*

Recombinant MPK3 (3.2 μg) and MPK6 proteins (5.7 μg) were activated by incubation with recombinant MKK5^DD^ (0.36 μg) in the presence of 50 μM ATP in 50 μl of reaction buffer (20 mM Hepes, pH 7.5, 10 mM MgCl_2_, and 1 mM DTT) at 25°C for 1 h. The activated MPK3 and MPK6 proteins were then used to phosphorylate the AtDOF4.7 protein (1.86 μg, 1:16 enzyme:substrate ratio) in the same reaction buffer containing 25 μM ATP and [γ-^32^P] ATP at 25°C for 30 min, according to Liu and Zhang (2014). The reaction was stopped by the addition of SDS gel-loading buffer. Using the Mini-PROTEAN^®^ Tetra System (Bio-Rad, USA), the samples were separated via electrophoresis on a 10% SDS-polyacrylamide gel at a constant 120 V. The gel was then dried under vacuum, and the phosphorylated AtDOF4.7 was visualized through autoradiography. Primary anti-His (TIANGEN, China) and anti-Flag (Cell Signaling Technology, USA) antibodies were used to verify the presence of the corresponding proteins in the phosphorylation buffer. Pre-stained protein markers (Fermentas, USA) were employed to calculate the molecular masses of the phosphorylated proteins.

## Results

### Ethylene Regulates the Expression of *AtDOF4.7*

A previous study indicated that overexpression of *AtDOF4.7* induces abscission defects in an ethylene-independent manner, although the flowers can perceive ethylene, suggesting that *AtDOF4.7* might not be a direct target of early ethylene-independent signal transduction (Wei et al., 2010). To investigate the role of *AtDOF4.7* downstream of the early abscission pathway, we examined the expression pattern of *Promoter_AtDOF4.7_::GUS* in the mutants with ethylene-insensitive and ethylene-independent abscission deficiencies.

**FIGURE 1:**
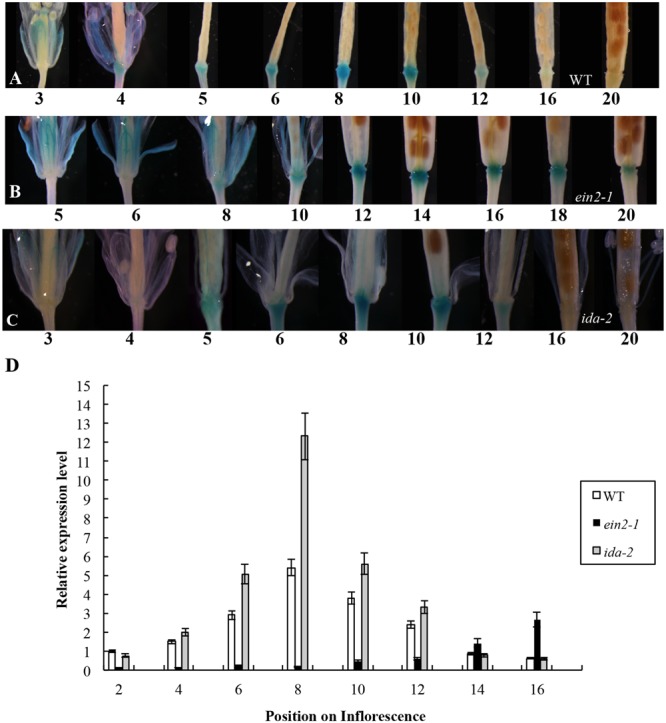
**Time-course of *Promoter_AtDOF4.7_::GUS* expression in WT, *ein2-1*, and *ida-2* flower AZ cells. (A)** *Promoter_AtDOF4.7_::GUS* expression in *A. thaliana* Col-0 (WT). *GUS* expression was first observed at flower position 4, and reached the maximum level of expression from positions 6 to 8. After flower position 10, expression was reduced, and no expression was detected from positions 16 to 20. Four-weeks-old plants were used for *GUS* staining. **(B)** *Promoter_AtDOF4.7_::GUS* expression in the *ein2-1* mutant. *GUS* staining was first observed at flower position 8, and accumulated to its maximum level from positions 12 to 14. After position 16, *GUS* staining declined, and little staining was observed at position 20. **(C)** *Promoter_AtDOF4.7_::GUS* expression in the *ida-2* mutant. The expression pattern was similar to that seen in WT. **(D)** Relative expression of *AtDOF4.7* at different flower positions in WT, *ein2-1* and *ida-2* plants. Flower position 2 in WT was the control. The temporal expression pattern of *AtDOF4.7* in WT was the same as in *ida-2*. However, the relative expression of *AtDOF4.7* in *ida-2* was higher from positions 6 to 10 than in WT. At flower positions 14 and 16, the expression level of *AtDOF4.7* in the *ida-2* background was similar to WT. Compared with WT, the temporal expression pattern of *AtDOF4.7* was delayed in the *ein2-1* mutant. The relative expression level of *AtDOF4.7* in *ein2-1* from positions 2 to 12 was significantly lower than in WT. After position 12, the relative expression level of *AtDOF4.7* was elevated >2-fold in the *ein2-1* compared with WT. Relative mRNA levels were averaged over three biological replicates and are shown with the SD (error bars).

In the earlier report, the first flower showing visible white petals was referred as flower position 1 and then counted downward along the inflorescence in sequence (Wei et al., 2010). We initially crossed a plant carrying a single-copy of the *Promoter_AtDOF4.7_::GUS* construct with *A. thaliana* Col-0 (wild-type). The *GUS* expression pattern in the *Promoter_AtDOF4.7_::GUS* plant was similar to that reported previously (**Figure 1A**). *GUS* expression was observed in the AZ cells at flower position 4, while at position 20, *GUS* expression from the *AtDOF4.7* promoter could scarcely be detected in the AZ cells.

The *Promoter_AtDOF4.7_::GUS* plants were then crossed with the ethylene-insensitive mutant *ein2-1*. The expression pattern of *Promoter_AtDOF4.7_::GUS* in the *ein2-1* mutant was altered temporally (**Figures 1B,D** and Supplementary Figure S3B), although spatial expression was similar to that of the wild type (WT). *GUS* expression was first observed at position 8 and continued to accumulate until reaching its maximum level at position 14. At position 20, *GUS* expression was still detectable in the AZ cells (**Figure 1B**).

The expression pattern of *Promoter_AtDOF4.7_::GUS* in the *etr1-1* mutant was also examined, and we found that the temporal expression pattern was altered in this mutant as well (Supplementary Figure S1). *GUS* expression was detectable at later flower positions than in *Promoter_AtDOF4.7_::GUS/ein2-1* flowers and was faintly visible at position 10. Expression was clearly observed at position 20. In addition, the profile of the spatial expression of *AtDOF4.7* in the *etr1-1* mutant was similar to that in *ein2-1* (**Figure 1B** and Supplementary Figure S1).

Temporally, the expression of *Promoter_AtDOF4.7_::GUS* in AZ cells in the ethylene-insensitive *ein2-1* and *etr1-1* mutants was remarkably delayed compared with that in WT. Because, flower abscission is also delayed in the inflorescences of the *ein2-1* and *etr1-1* mutants (Butenko et al., 2003; Patterson and Bleecker, 2004), we concluded that ethylene can influence *AtDOF4.7* expression. To confirm this finding, we placed transgenic plants expressing *Promoter_AtDOF4.7_::GUS* in an air-tight chamber containing 10 ppm ethylene gas, which can accelerate organ abscission. Compared with the *Promoter_AtDOF4.7_::GUS* plants that were exposed to air (in the absence of ethylene), *GUS* expression was detected earlier at flower position 1 in the presence of ethylene (Supplementary Figure S2). The time-course of *Promoter_AtDOF4.7_::GUS* expression was shortened by ethylene treatment. These experimental results indicated that ethylene can influence the timing of *AtDOF4.7* expression, and the time-course of *AtDOF4.7* expression could be relevant to organ abscission.

### *AtDOF4.7* Is a Component of the *IDA*-Mediated Abscission Pathway Underlying Abscission

In the *IDA*-mediated abscission pathway in *Arabidopsis*, IDA binds as a ligand to the RLKs HAE and HSL2 (Stenvik et al., 2008). Some cytoplasmic effectors, such as MKK4, MKK5, MPK3, and MPK6, function together to control cell separation during abscission (Cho et al., 2008). As reported previously, IDA can control the intensity of organ abscission (Butenko et al., 2003; Stenvik et al., 2006). To further determine whether the expression of *AtDOF4.7* is regulated by *IDA* at the transcriptional level, we crossed a *Promoter_AtDOF4.7_::GUS* transgenic plant with the *ida-2* (Col-0) mutant. The temporal expression pattern of *Promoter_AtDOF4.7_::GUS* was unaltered in the AZ cells of the siliques in the *ida-2* background and was similar to that of *AtDOF4.7* in the WT background (**Figures 1A,C**). However, qRT-PCR showed that the relative expression levels of *AtDOF4.7* from flower positions 6 to 10 were significantly higher in the *ida-2* background that in WT (**Figure 1D** and Supplementary Figure S3A). These data suggested that *IDA* negatively regulates the expression of *AtDOF4.7* at the transcriptional level.

**FIGURE 2:**
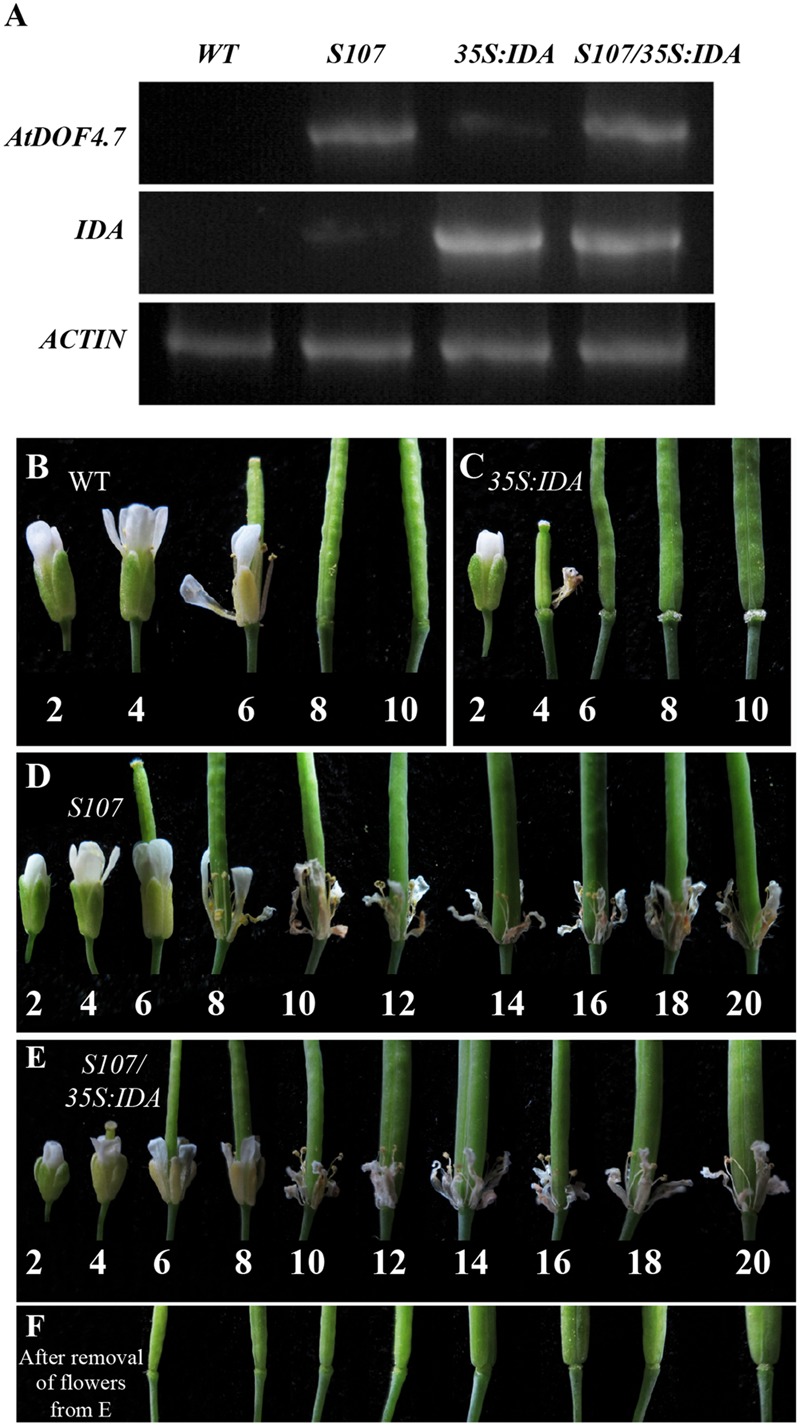
***S107/35S:IDA* plants are deficient in floral organ abscission. (A)** RT-PCR analysis of *AtDOF4.7* and *IDA* expression in *S107/35S:IDA* transgenic flowers. *ACTIN2* (*At3g18780*) was used as the internal control gene. Both *AtDOF4.7* and *IDA* were overexpressed in *S107/35S:IDA* siliques. **(B)** Normal abscission phenotype in WT. Organ abscission began at position 6 and ended at position 8. **(C)** Premature abscission at position 4 in *35S:IDA* flowers. After abscission, white substrate AGPs accumulated around AZ cells at position 6 and, apparently, at position 10. **(D)** Organ abscission defects from positions 2 to 20 in *S107* flowers. **(E)** Organ abscission defects in *S107/35S:IDA* flowers from positions 2 to 20. **(F)** After removal of the petals and filaments in **(E)** from positions 6 to 20, no AGPs were detected around the AZ cells in *S107/35S:IDA* siliques. The abscission phenotypes of 4-weeks-old plants are shown in **(B–E)**.

To confirm the hypothesis that *IDA* and *AtDOF4.7* are involved in a common pathway, we crossed an *AtDOF4.7*-overexpressing line (*S107*) with *35S:IDA* in the Col-0 background, which shows early abscission of the flowers at position 3 (Stenvik et al., 2006). Semi-quantitative RT-PCR demonstrated that both the *AtDOF4.7* and *IDA* genes were overexpressed in the siliques of *S107/35S:IDA* plants (**Figure 2A**). The *S107/35S:IDA* lines showed that the floral organ abscission defect phenotype was similar to that in *S107* plants (**Figures 2D,E**). Most notably, although the transcript levels of *IDA* were higher than that of *AtDOF4.7* in *S107/35S:IDA* lines, from which the floral organs could not shed (**Figures 2A,E**). This data suggested that *AtDOF4.7* might be epistatic to *IDA*. The flowers of WT plants exhibited normal abscission at position 6 (**Figure 2B**). After abscission beginning at position 6, excessive secretion of arabinogalactan protein (AGP) was observed in the AZ cells of the flowers of *35S:IDA* plants (Stenvik et al., 2006; **Figure 2C**). However, there was no AGP observed in the AZ cells of siliques of *S107/35S:IDA* plants, even after the floral parts on the fruits were removed from positions 6 to 20 (**Figure 2F**). These results support the hypothesis that *IDA* and *AtDOF4.7* act in a common pathway to regulate abscission, and *IDA* might be in the upstream abscission pathway.

### Interaction of AtDOF4.7 with MPK3 and MPK6 *In Vivo* and *In Vitro*

**FIGURE 3:**
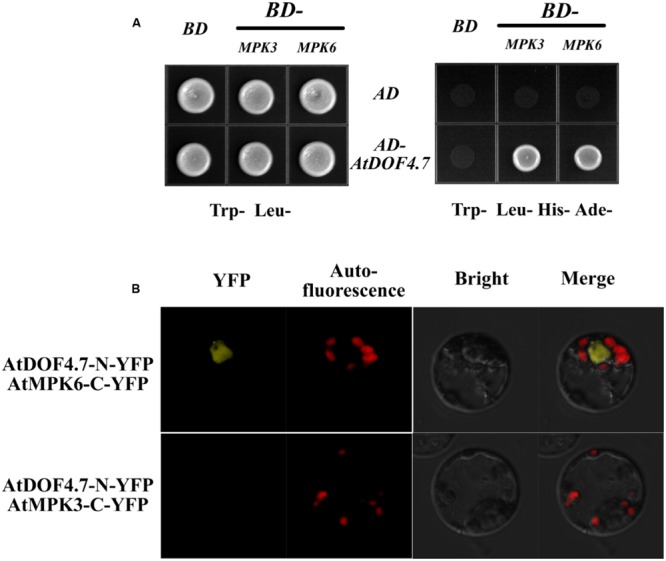
**Interaction of AtDOF4.7 with MPK3 and MPK6 *in vivo*. (A)** Yeast two-hybrid (Y2H) assay. Although yeast cells containing the indicated vectors could grow on Trp*^-^* Leu***^-^*** medium, only cells containing the *pAD-GAL4-AtDOF4.7* and *pBD-GAL4-MPK3/MPK6* expression vectors could grow on medium lacking Trp, Leu, His, and Ade. **(B)** Bifluorescence complementation (BiFc) assay. This assay showing the colocalization of *pUC-SPYNE-AtDOF4.7* with *pUC-SPYCE-MPK6* in the nuclei of mesophyll protoplasts. Whereas, fluorescence was not be observed from mesophyll protoplasts containing *pUC-SPYNE-AtDOF4.7* and *pUC-SPYCE-MPK3*.

A previous study found that the *MPK6^KR^/mpk3* mutant, but not *mpk3* or *mpk6* single mutants, showed a defective floral abscission phenotype (Cho et al., 2008). Because MPK3 and MPK6 are key components of the *IDA*-mediated pathway underlying abscission, to understand how *AtDOF4.7* is regulated by the *MAPKs*, we performed yeast two-hybrid (Y2H) and BiFC assays to determine whether AtDOF4.7 interacts with MPK3 or MPK6. The Y2H experiment showed that the transformed yeast cells were able to grow on 5 mM 3-AT medium lacking Trp, Leu, His, and Ade, suggesting an interaction between *pAD-GAL4-AtDOF4.7* and *pBD-GAL4-MPK3/MPK6 in vivo* (**Figure 3A**). To further confirm the interaction of AtDOF4.7 with MPK3 or MPK6, we constructed the BiFC vectors *pUC-SPYNE-AtDOF4.7* and pUC-SPYCE-MPK3/6, and the two constructs were co-transformed into *Arabidopsis* mesophyll protoplasts. The cells were then observed using laser fluorescence confocal microscopy; which showed that *pUC-SPYNE-AtDOF4.7* and pUC-SPYCE-MPK6 interact in the nuclei (**Figure 3B**). However, the yellow fluorescence could not be detected from the cells containing *pUC-SPYNE-AtDOF4.7* and pUC-SPYCE-MPK3. The BiFC results strongly suggested that AtDOF4.7 and MPK6 physically interact in *Arabidopsis*.

### Phosphorylation of AtDOF4.7 by MPK3 and MPK6 *In Vitro*

**FIGURE 4:**
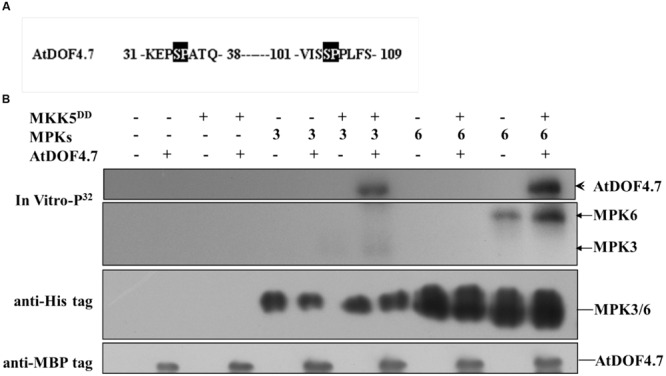
**Phosphorylation of AtDOF4.7 by activated MPK3 and MPK6 *in vitro*. (A)** Partial sequences of the AtDOF4.7 protein. Black boxes indicate the conserved MAPK phosphorylation sites; **(B)** MPK3 and MPK6 phosphorylate the MBP-tagged AtDOF4.7 recombinant protein *in vitro*. The arrowhead indicates the phosphorylated AtDOF4.7 protein. Bands indicated by the arrows are MPK3 or MPK6 phosphorylated by MKK5^DD^. Anti-His and anti-MBP tag primary antibodies were used to detect the corresponding proteins in the phosphorylation buffer.

Potential sites for MAPK phosphorylation are Ser/Thr residues followed by a Pro residue (Meng et al., 2013). There are two such sites in the *AtDOF4.7* sequence: Ser-34-Pro and Ser-104-Pro (**Figure 4A**). To determine whether MPK3/MPK6 is able to carry out phosphorylation of AtDOF4.7, we obtained the recombinant proteins MBP-tagged AtDOF4.7, His_6_-tagged MPK3 and MPK6, and FLAG-tagged MKK5^DD^ for phosphorylation assays. Following activation by purified FLAG-tagged recombinant MKK5^DD^, either His_6_-tagged MPK3 or MPK6 strongly phosphorylated AtDOF4.7 (**Figure 4B**).

### Constitutively Activated *MKK5* Cannot Rescue Organ Abscission in the *S107* Line

**FIGURE 5:**
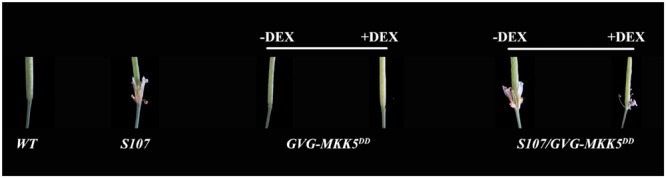
***AtDOF4.7* is involved in abscission mediated by the *MAPK* cascade.** The defective abscission defective phenotype of *S107* was partly restored in *S107/GVG-MKK5^DD^* plants following DEX treatment. All representative siliques are from flower position 10 of WT, *S107, GVG-MKK5^DD^*, and *S107/GVG-MKK5^DD^* plants.

MKK4 and MKK5 are homologs found in *Arabidopsis* that act upstream of MPK3 and MPK6 in the regulation of floral abscission (Cho et al., 2008). Plants expressing the constitutively activated forms of *MKK4* or *MKK5* under the control of a steroid-inducible promoter (designated *GVG-MKK4^DD^* or *GVG-MKK5^DD^*) had a normal abscission phenotype (Ren et al., 2002; Cho et al., 2008). To assess the biological function of the phosphorylation of AtDOF4.7 by MAPK, we crossed the *AtDOF4.7*-overexpressing *S107* line with a *GVG-MKK5^DD^* transgenic plant to obtain *S107/MKK5^DD^*. We examined the siliques of *S107/MKK5^DD^* plants at flower position 10, which is the position at which WT flowers shed completely. We observed that the organ abscission phenotype of *S107/MKK5^DD^* was not rescued; however, small parts of the flowers were shed from the siliques of *S107/MKK5^DD^* plants after dexamethasone (DEX) treatment (**Figure 5**). These data indicated that the two genes act in a common pathway to regulate abscission in *Arabidopsis*, and *MKK5* may negatively regulate *AtDOF4.7*.

**FIGURE 6:**
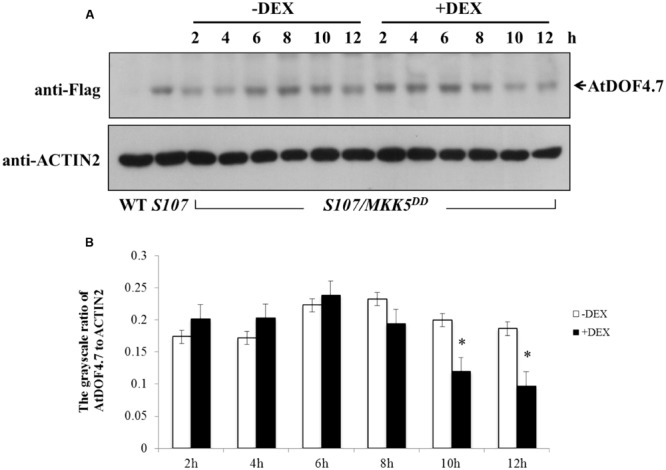
**Determination of AtDOF4.7 protein levels in *S107/MKK5^DD^* siliques. (A)** Without DEX treatment, the AtDOF4.7 protein levels did not change apparently over the 12 h time courses of the experiment. However, the level of the AtDOF4.7 protein appeared to be greatly decreased at the 10 and 12 h time points following DEX treatment. The bands indicated by arrows are the AtDOF4.7 protein. An anti-ACTIN2 antibody was used as the internal control. Total protein extracts from WT and *S107* were used as the negative and positive controls, respectively. **(B)** The grayscale ratio of these bands without and with DEX treatment from **(A)**. The asterisks indicate significant differences between DEX treatment and the control (-DEX) at the 10 and 12 h time points (*t*-test, *P* < 0.05). Grayscale measurements of each band were repeated three times.

A phosphorylation-dependent mechanism is involved in protein degradation (Willems et al., 1999; Feng et al., 2004; Yoo et al., 2008). Therefore, a western blot assay was performed to determine the changes in AtDOF4.7 protein levels in *S107/MKK5^DD^* plants treated with DEX. The siliques of *S107/MKK5^DD^* at flower position 10 were induced with 15 μM DEX after 10 h, resulting in greatly decreased AtDOF4.7 protein levels (**Figure 6A**). The grayscale ratio of AtDOF4.7 protein bands on the western blot were measured, and the results showed that the changes in the levels of the AtDOF4.7 protein exhibited significant differences after 10 and 12 h of DEX treatment compared with the untreated control (**Figure 6B**). ACTIN2 was used as an internal protein control, and was detected with an anti-ACTIN2 antibody. These data suggest that activated MKK5 following DEX induction could affect AtDOF4.7 at the protein level in the *S107/MKK5^DD^* plant, providing one explanation for how organ abscission is partially recovered in *S107/MKK5^DD^* lines.

## Discussion

A recent report shows that ethylene manipulates the rate of abscission, rather than directly inducing it (Niederhuth et al., 2013a). It has been indicated that several ethylene mutants of *Arabidopsis*, such as those harboring the ethylene receptor ethylene-response 1 (*etr1*) and ethylene-insensitive 2 (*ein2*) point mutations, displayed a delay of floral organ abscission (Bleecker et al., 1988; Guzman and Ecker, 1990; Patterson and Bleecker, 2004), however, the formation and differentiation of the AZ cells are not affected in these ethylene-insensitive mutants. Up to now, the genetic mechanism through which ethylene regulates abscission is still unclear. Here, we suggest that ethylene accelerates organ abscission in *Arabidopsis* by regulating the time-course of the expression of *AtDOF4.7* (**Figure 1B**, Supplementary Figures S1, S2, and S3B). To make a preliminary demonstration for this hypothesis, we analyzed the region 3000 bp upstream of the *AtDOF4.7* promoter sequence and found that the promoter sequence of *AtDOF4.7* contained three *cis*-acting elements in this region: a GCC box (-1702 to -1696 bp), a CRT/DRE element (-1029 to -1023 bp) and several EIN3-binding sites (EBS, ATGTA; Supplementary Figure S4). The first two elements can be recognized by Ethylene Response Factors (ERFs) in response to ethylene (GCC) and drought stress (CRT/DRE), while the last element can be recognized by EIN3. Most notably, in the ethylene signaling pathway, ERFs are critical factors responding to ethylene, which can be activated by EIN3 (Ohme-Takagi and Shinshi, 1995; Chao et al., 1997). Based on these observations, *AtDOF4.7* might be a potential target gene of ERFs or EIN3 in response to ethylene. From the data presented in this study, we hypothesize that the regulation of *AtDOF4.7* is related to ethylene, but it is unclear that how ethylene affecting the expression of *AtDOF4.7* at the transcriptional level.

Auxin, as an antagonist of ethylene, negatively regulates the cell wall-degrading enzymes, leading to a delay in cell separation via an ethylene-independent mechanism. Additionally, the AZ patterning is not affected in *arf2* mutants (Ellis et al., 2005). It has been reported that auxin may regulate *IDA* and *HAE/HSL2* to control cell separation (Kumpf et al., 2013; Niederhuth et al., 2013a). Thus, we can put forward the hypothesis that both ethylene and auxin may be involved in the regulation of *AtDOF4.7* expression.

Previous data indicated that the delay of abscission caused by *AtDOF4.7* overexpression could not be accelerated by exogenous ethylene, suggesting the potential involvement of ethylene-independent regulation. Therefore, we investigated the relationship between *IDA* and *AtDOF4.7* in this study. In the mutant of *IDA*, the expression pattern of *AtDOF4.7* promoter was not significantly altered (**Figure 1C**), while the transcript level of *AtDOF4.7* was found to be up-regulated at the same flower positions in the *ida-2* mutant (**Figure 1D**). *IDA* is involved in the regulation of *ADPG2* expression in the AZ to control cell separation during floral organ abscission (González-Carranza et al., 2002, 2007; Ogawa et al., 2009; González-Carranza et al., 2012), and its expression is suppressed by overexpression of *AtDOF4.7* in the regulation of abscission (Wei et al., 2010). Our results indicated that the overexpression of *AtDOF4.7* could inhibit the early abscission caused by *IDA* overexpressing lines (**Figure 2**). Therefore, this result provides a hint that *AtDOF4.7* might participate in the regulation of *PGs* by *IDA*. It has been well-established that MAPK cascades (mainly MKK4/MKK5-MPK3/MPK6 pathway) are involved in downstream of *IDA*-mediated abscission pathway. Thus, the relations between the *MAPK*s and *AtDOF4.7* were also investigated. First, we found two abscission-related MPKs, MPK3 and MPK6, could interact with AtDOF4.7 in yeast. The *in vitro* assay indicated that both MPK3 and MPK6 could phosphorylated AtDOF4.7, and the signal of MPK6 was apparently stronger than that of MPK3. This is consistent with the stronger *in vivo* interaction between MPK6 and AtDOF4.7, suggesting MPK6 may play more important role in the regulation of abscission. Second, our results indicated that the *AtDOF4.7*-induced abortion of abscission could be partially rescued by MKK activation (**Figure 5**), and the protein level of AtDOF4.7 is down-regulated following the MKK5 activation. Thus, it is reasonable to speculate that the AtDOF4.7 may act as a direct target of MPKs in the abscission regulation. Taken together, our results suggested that the *AtDOF4.7* should also involved in downstream of the *IDA*-*MAPK*-mediated abscission pathway.

## Conclusion

*AtDOF4.7*, as a negative regulator in floral organ abscission, is regulated by the ethylene-dependent and ethylene-independent abscission pathways. It is suggested that *AtDOF4.7* might be regulated by ethylene, and also by *IDA*, which represses expression of *AtDOF4.7*. Nevertheless, the relationship between phosphorylation and ethylene signaling remains unclear. A previous study showed that phosphorylated MPK3/6 activated by MKK4/5 further phosphorylates ERF6 to regulate defense gene induction and fungal resistance ((Meng et al., 2013). Therefore, it is worthwhile to explore whether the phosphorylation of ERFs by MPK3/6 in the regulation of *AtDOF4.7* occurs in response to ethylene. Further detailed biochemical and genetic analyses will allow us to gain a better understanding of the importance of AtDOF4.7 phosphorylation and ethylene responses in plant organ abscission.

## Author Contributions

G-QW, P-CW, FT, MY, and X-YZ performed the research; G-QW, P-CW, FT, Q-JC, and X-CW analyzed the data; G-QW, P-CW, and X-CW designed the research, G-QW and X-CW wrote the article with contributions from G-QW, P-CW, FT, and MY.

## Conflict of Interest Statement

The authors declare that the research was conducted in the absence of any commercial or financial relationships that could be construed as a potential conflict of interest.
