# Supplementary material for: The Transcription Factor AtDOF4.7 Is Involved in Ethylene- and IDA-Mediated Organ Abscission in Arabidopsis
Source: Front Plant Sci. 2016 Jun 17;7:863. doi: 10.3389/fpls.2016.00863 (PMC4911407; doi:10.3389/fpls.2016.00863)
Supplement: Supplementary file 1 [file Table_1.DOC]

***SUPPLEMENTARY MATERIAL***

**The Transcription Factor AtDOF4.7 is Involved in Ethylene- and IDA- mediated Organ Abscission in *Arabidopsis***

Gao-Qi Wang, Peng-Cheng Wei, Feng Tan, Man Yu, Xiao-Yan Zhang, Qi-Jun Chen, and Xue-Chen Wang*

***Correspondence**: Xue-Chen Wang [xcwang@cau.edu.cn](mailto:xcwang@cau.edu.cn)

**Supplementary Table S1.** DNA sequencing primers used to characterize hybrid plants in *Arabidopsis* crosses.

| Primer name | Sequence (5’ to 3’) | Note |
| --- | --- | --- |
| 47PN  GUSRP  *IDA*LP  *IDA*RP  LB1.3  *35S*FD3  *IDA*RP  *SUPER*FP  *AtDOF47*RP  *GVG*FP  *GVG*RP | GTGATTGTGTTATTGTGTCTA  TCACCGAAGTTCATGCCAGTCCAG  TTTTGGCCACTTGAGAAATTG  GAAAATAAAAGTCGAAGGCGG  ATTTTGCCGATTTCGGAAC  GATGACGCACAATCCCACTATCCTT  CTTAGAAGGAGCAGAAGGAGGAATG  AGAAATGGATAAATAGCCTTGC  GAGCAGAGCATTATTATTAGC  GAACAACTGGGAGTGTCG  TTGGCTCTTCAGACCTTCCTTA | Genotyping *pAtDOF4.7::GUS*  Genotyping *ida*  Genotyping *35S:IDA*  Genotyping *S107*  Genotyping *GVG-MKK5^DD^* |
