# Supplementary material for: The Transcription Factor AtDOF4.7 Is Involved in Ethylene- and IDA-Mediated Organ Abscission in Arabidopsis
Source: Front Plant Sci. 2016 Jun 17;7:863. doi: 10.3389/fpls.2016.00863 (PMC4911407; doi:10.3389/fpls.2016.00863)
Supplement: Supplementary file 2 [file Table_2.DOC]

***SUPPLEMENTARY MATERIAL***

**The Transcription Factor AtDOF4.7 is Involved in Ethylene- and IDA- mediated Organ Abscission in *Arabidopsis***

Gao-Qi Wang, Peng-Cheng Wei, Feng Tan, Man Yu, Xiao-Yan Zhang, Qi-Jun Chen, and Xue-Chen Wang*

***Correspondence**: Xue-Chen Wang xcwang@cau.edu.cn

**Supplementary Table S2.** Oligonucleotide primers used for semi-quantitative PCR and quantitative real-time PCR (qRT-PCR).

| Primer name | Sequence (5’ to 3’) | Primer name | Sequence (5’ to 3’) |
| --- | --- | --- | --- |
| *ACTIN-*semiF  *ACTIN-*semiR  *AtDOF4.7*-semiF  *AtDOF4.7*-semiR  *IDA*-semiF  *IDA*-semiR | CATCAGGAAGGACTTGTACGG  GATGGACCTGACTCGTCATAC  GCAGCCTCCAAGTCATCT  TCCAGTGGCGAACCTATC  CAAATGGCTCCGTGTCGT  TCAATGAGGAAGAGAGTTAACA | *ACTIN2/8* realF  *ACTIN2/8* realR  *AtDOF4.7* realF  *AtDOF4.7* realR  IDA realF  IDA realR | GGTAACATTGTGCTCAGTGGTGG  AACGACCTTAATCTTCATGCTGC  CTCGTGAGCTTGTAAGAAACCA  GAGGCAAGGTTGAAGTTAGGAT  TGTGTAGCGGCTGCAAGAATTG  AGGAGGAATGGGAACGCCTTTA |
