# Supplementary material for: The Transcription Factor AtDOF4.7 Is Involved in Ethylene- and IDA-Mediated Organ Abscission in Arabidopsis
Source: Front Plant Sci. 2016 Jun 17;7:863. doi: 10.3389/fpls.2016.00863 (PMC4911407; doi:10.3389/fpls.2016.00863)
Supplement: Supplementary file 3 [file Image_1.PDF]

SUPPLEMENTARY MATERIAL

The Transcription Factor AtDOF4.7 is Involved in Ethylene- and IDA- mediated Organ Abscission in *Arabidopsis*

Gao-Qi Wang, Peng-Cheng Wei, Feng Tan, Man Yu, Xiao-Yan Zhang, Qi-Jun Chen, and Xue-Chen Wang*

***Correspondence**: Xue-Chen Wang xcwang@cau.edu.cn

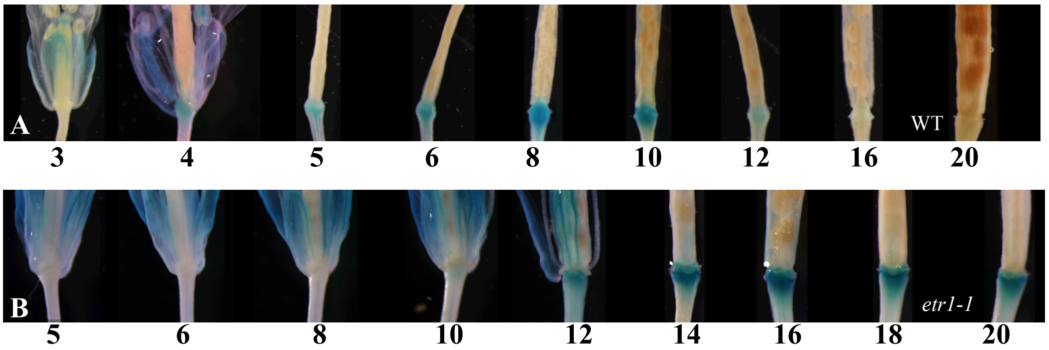

Supplementary Figure S1. Time-course experiment showing the expression of the *Promoter*$_{AtDOF4.7}$::*GUS* construct in the AZ cells of flowers and siliques in the *etr1-1* mutant background.

(A) Time-course of Promoter$_{AtDof4.7}$::*GUS* expression in WT Col-0. *GUS* staining was first observed at flower position 4, and its expression declined after flower position 10, with no staining being observed at positions 16 and 20;

(B) Time-course of Promoter$_{AtDof4.7}$::*GUS* expression in the *etr1-1* mutant background; *GUS* staining was first observed at flower position 10; the maximum levels of expression occured at positions 14 to 16; and the relative level of *GUS* expression remained high at positions 18 and 20.
