# Supplementary material for: The Transcription Factor AtDOF4.7 Is Involved in Ethylene- and IDA-Mediated Organ Abscission in Arabidopsis
Source: Front Plant Sci. 2016 Jun 17;7:863. doi: 10.3389/fpls.2016.00863 (PMC4911407; doi:10.3389/fpls.2016.00863)
Supplement: Supplementary file 4 [file Image_2.PDF]

SUPPLEMENTARY MATERIAL

The Transcription Factor AtDOF4.7 is Involved in Ethylene- and IDA- mediated Organ Abscission in *Arabidopsis*

Gao-Qi Wang, Peng-Cheng Wei, Feng Tan, Man Yu, Xiao-Yan Zhang, Qi-Jun Chen, and Xue-Chen Wang*

***Correspondence**: Xue-Chen Wang   xcwang@cau.edu.cn

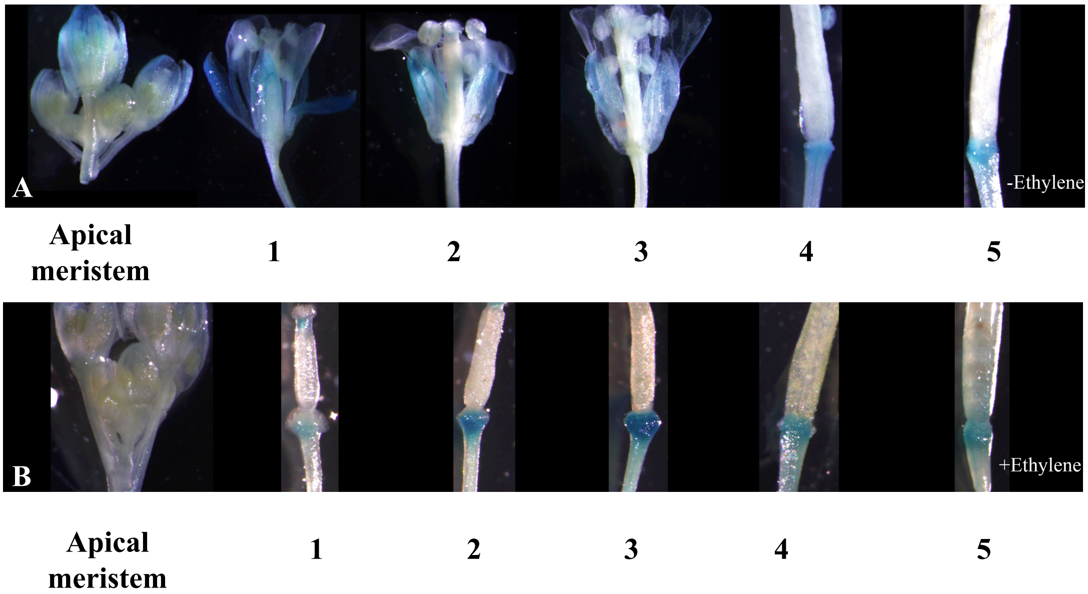

Supplementary Figure S2. Time-course of the expression of the *Promoter*$_{AtDOF4.7}$::*GUS* construct in the AZ cells of flowers and siliques after treatment with 10 ppm ethylene gas.

Time-course of *Promoter*$_{AtDOF4.7}$::*GUS* expression in transgenic lines exposed to air. *GUS* staining was first observed in the AZ cells at flower position 4. (B) Time-course of *Promoter*$_{AtDOF4.7}$::*GUS* expression in transgenic lines exposed to 10 ppm ethylene. GUS staining was faintly visible in the AZ cells of the apical meristem, and visible GUS staining was detectable beginning at flower position 1.
