# Supplementary material for: The Transcription Factor AtDOF4.7 Is Involved in Ethylene- and IDA-Mediated Organ Abscission in Arabidopsis
Source: Front Plant Sci. 2016 Jun 17;7:863. doi: 10.3389/fpls.2016.00863 (PMC4911407; doi:10.3389/fpls.2016.00863)
Supplement: Supplementary file 5 [file Image_3.PDF]

SUPPLEMENTARY MATERIAL

The Transcription Factor AtDOF4.7 is Involved in Ethylene- and IDA- mediated Organ Abscission in *Arabidopsis*

Gao-Qi Wang, Peng-Cheng Wei, Feng Tan, Man Yu, Xiao-Yan Zhang, Qi-Jun Chen, and Xue-Chen Wang*

***Correspondence**: Xue-Chen Wang xcwang@cau.edu.cn

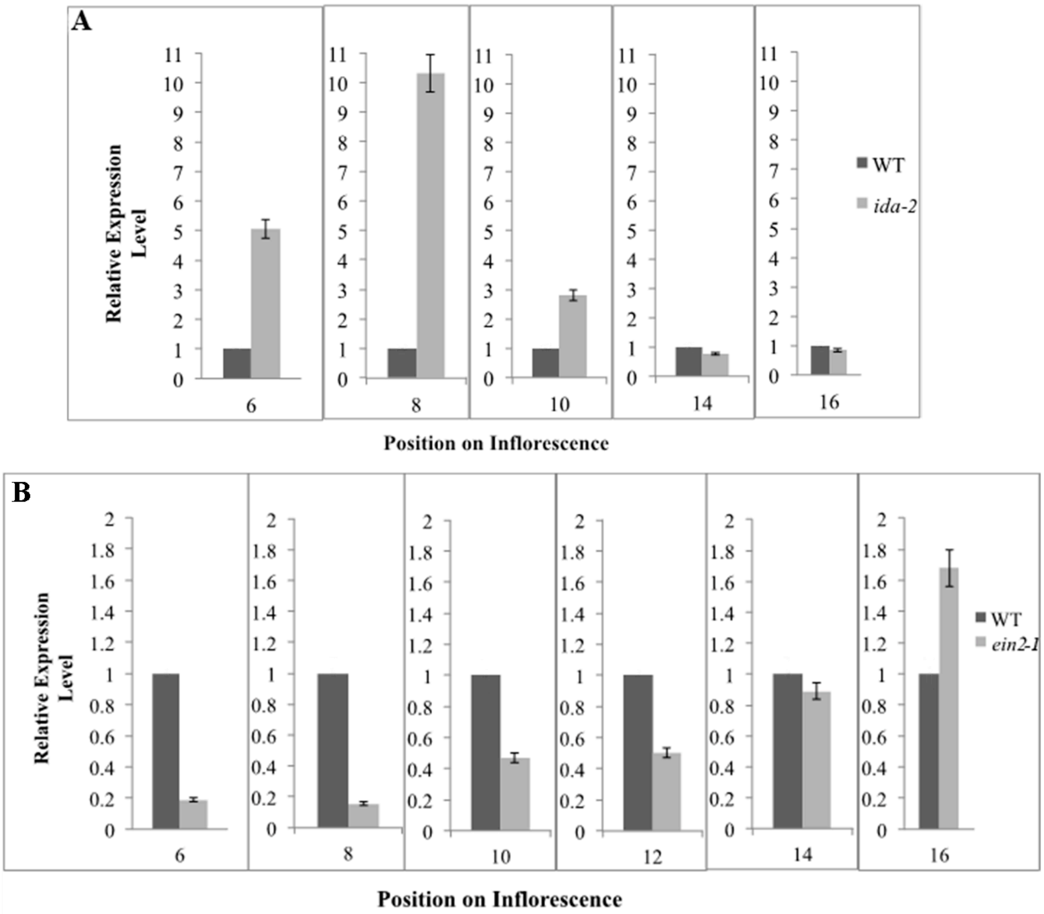

Supplementary Figure S3. Relative expression of *AtDOF4.7* at different flower positions in the *ida-2* and *ein2-1* mutant backgrounds.

(A) qRT-PCR analysis of *AtDOF4.7* at different flower positions in 4-week-old *ida-2* mutant plants. The expression level of *AtDOF4.7* was 5-fold higher at position 6, and 10-fold higher at position 8 than in WT. At position 10, the expression level was reduced, but was still slightly higher in the *ida-2* than in WT. At flower positions 14 and 16, the expression level of *AtDOF4.7* in the *ida-2* mutant was similar to that in WT. (B) Compared with WT, the relative expression level of *AtDOF4.7* was lower in the *ein2-1* mutant from flower positions 6 to 12. At position 14, the relative expression level was almost equal to that of WT. At flower position 16, however, expression was higher in *ein2* because *AtDOF4.7* was not expressed after flower position 16 in WT. The relative mRNA levels were averaged over three biological replicates and are shown with the SD (error bars).
