# Supplementary material for: The Transcription Factor AtDOF4.7 Is Involved in Ethylene- and IDA-Mediated Organ Abscission in Arabidopsis
Source: Front Plant Sci. 2016 Jun 17;7:863. doi: 10.3389/fpls.2016.00863 (PMC4911407; doi:10.3389/fpls.2016.00863)
Supplement: Supplementary file 6 [file Image_4.PDF]

SUPPLEMENTARY MATERIAL

The Transcription Factor AtDOF4.7 is Involved in Ethylene- and IDA- mediated Organ Abscission in *Arabidopsis*

Gao-Qi Wang, Peng-Cheng Wei, Feng Tan, Man Yu, Xiao-Yan Zhang, Qi-Jun Chen, and Xue-Chen Wang*

***Correspondence**: Xue-Chen Wang   xcwang@cau.edu.cn

```
-1810  GTACGGATTC CCGTACTACG ACAACACCAT AACATACGGT GGTTACTCCG ACCACATGGT TTGCGAGGAA
-1740  GGTTTCGTCA TCCGTATTCC AGACAATCTC CCCTTGGACG CCGCCGCACC GCTCCTCTGT GCCGGTATCA
-1670  CGGTCTATTC CCCTATGAAG TATCACGGGC TCGACAAACC CGGTATGCAC ATCGGTGTGG TAGGATTAGG
-1600  CGGTTTAGGT CATGTAGGAG TGAAATTTGC CAAGGCTATG GGTACTAAGG TTACGGTTAT TAGTACTTCG
-1530  GAGAAAAAGA GAGATGAGGC GATTAATCGG CTTGGTGCGG ATGCTTTCTT GGTGAGCCGT GACCCAAAAC
-1460  AGATTAAGGA TGCAATGGGT ACTATGGATG GTATAATTGA TACCGTCTCT GCGACTCATT CACTTCTTCC
-1390  GTTGCTCGGT TTGCTGAAGC ATAAGGGAAA ACTTGTTATG GTTGGTGCAC CCGAGAAGCC ACTCGAGCTA
-1320  CCTGTCATGC CTCTCATCTT TGGTAAGCTA TATTTGTAGT AACAAATCCG TATATATATG ACTTGTGCTA
-1250  AATTTGCAAT GTTTAGTCAA TAACGTGTGA AAATAACATT GAAATTGTGT GTGATTGTGT TATTGTGTCT
-1180  ATATAGAGAG GAAGATGGTA ATGGGAAGTA TGATAGGAGG GATAAAAGAG ACCCAGGAAA TGATAGATAT
-1110  GGCCGGGAAA CACAACATCA CTGCGGATAT TGAGCTTATC TCTGCCGATT ATGTCAACAC CGCCATGGAA
-1040  CGGCTAGAGA AAGCCGACGT TAGGTACCGC TTTGTGATTG ATGTTGCCAA CACATTGAAG CCTAATCCTA
 -970  ATTTATAAGT TTTAAGCATT AAACTTCCTC AAATGTTATT TTCTTGTTTG GTTTGTCGTA CTAATTAAGT
 -900  TTTGTGTTGT TGTATAACTA TATAATTTGG TATGCTTAAT CCAATAAATG AATAGTATGA TATATATGAT
 -830  AAGATTTTGG GGGTAAGCCA AGAGTTATAT ATTAGTATCT AAGGTTTGGA TAAAGAACAG TACTGTTAAA
 -760  TACCAAAAAT GTAAAGATGA TGAAAGAATA AAAATCATTT TACAGTGTGA AAAAAATCAG TTTATCATTA
 -690  GGTGAGTTAT CAACCTGAAA TTAGATTACC ATGTTCTCGA TCGTGTGATA AATATCTATC TTCATATTGG
 -620  ATGATGGAGC GATAGAAGTG AAAAGTCAAA GAGGATAATA AATGAACCGT ATACGACGAC TTGTGTTGTG
 -550  TTGCTTCCAA CAAGAGAGGG GACAGTGAAG TTCGAGTTTG TTCCCAATGC GAAAGCACTC CATTCCTTGA
 -480  TATGGCTTCT CTTTCTTTAC CTCGTACCTT TTACCACACT TCTCTCTCTC TCTCTCTCTC TCTCTCTCTC
 -410  TTCCCTAGGT CGATCTTATC TTGTCTTGTC TGAATATTTC ATAATATAAT GATACAAGAG ATTTTGCTTG
 -340  TAACATACGA ATGCGTATAT TATTTGAAAA TTCAAACTTA GGAACATGTT ACTGAAGGGA AGCTTTGCTC
 -270  GACTATATAT GTAAATGTTT TCTTATATAT ATTTCAAAAT CATATATATA TATATATATA TATTAATGTA
 -200  AATTTTTTAG TACTTTGTAA TTTAGATAGT TAAATGAACT TGGAAAACTC TATTAGACTA TACACTTGGT
 -130  AATTAGACAC ATTTGGTTTG AAAATATTTA TTAAAATATG CATATACTAA ACAACAAAGC ATGCATATGT
  -60  ACATATGTAT ATACACTTTT ATATACGTCA TTGTGTTCAT ATATATACAT TCGAAGATT C
```

Supplementary Figure S4. The upstream promoter region of the *AtDOF4.7* gene.

We analyzed the 3000 bp of the genomic DNA sequence upstream from the *AtDOF4.7* promoter. Two ERF *cis*-acting elements, a GCC box (indicated by a black box) and a CRT/DRE element (indicated by a gray box), as well as six EBS (EIN3-binding sites, ATGTA) elements (indicated by unshaded boxes) were detected. The conserved GCC box sequence is AGCCGCC, but the first G base, the fourth G base and the sixth C base are required for recognition by the ERF motif in response to ethylene. Therefore, the promoter sequence of *AtDOF4.7*, which contains the ACGCCGCC sequence, can be recognized by ERFs.
